# Effects of Continuous Electric/Magnetic Field Treatment on Nutrient, Enzyme Activity, and Bacterial Community Structure in Rocky Desertification Soils

**DOI:** 10.3390/microorganisms14040934

**Published:** 2026-04-21

**Authors:** Jun Hu, Yungen Liu, Yan Wang, Wenjiao Gao, Jiaxu Zhang, Silin Yang, Feifeng Deng, Bo Yang, Caishuang Huang

**Affiliations:** 1 School of Soil and Water Conservation, Southwest Forestry University, Kunming 650224, China; hujun@swfu.edu.cn (J.H.);; 2 Key Laboratory of Ecological Environment Evolution and Pollution Control in Mountainous & Rural Areas of Yunnan Province, Kunming 650224, China; 3 Zhanyi Karst Ecosystem Observation and Research Station, Qujing 655333, China

**Keywords:** rocky desertification, electric and magnetic field treatments, soil nutrients and enzyme activities, bacterial community

## Abstract

Soil nutrient loss and infertility in rocky desertification areas severely constrain ecological restoration. Exploring the impacts of external field remediation technologies on soil quality in these regions may offer novel strategies for soil enhancement and ecosystem recovery. This study conducted a three-month experiment to investigate the impact of continuous electric (ET, 20 V) and magnetic (MT, 200 mT) field treatments on soil nutrients, enzyme activities, and bacterial communities in simulated moderate and severe rocky desertification soils. Results showed that although an overall declining trends in total contents of key soil nutrients (Total nitrogen, total phosphorus, and total potassium), both electric and magnetic field treatments effectively mitigated the decreases of total nitrogen and potassium content (with the exception of total phosphorus) in rocky desertification soils, while improving their available contents compared to the control (CK). Electric field application significantly reduced the pH of moderate and severe rocky desertification soils through electrolysis, shifting the soil from alkaline (pH 7.69 and 7.73, respectively) to slightly acidic (pH 6.71 and 6.37, respectively); Both electric and magnetic field treatments enhanced urease and sucrase activities in moderately and severely rocky desertified soils. Compared to the CK, the increases were 21.92%, 4.46%, 5.70%, and 66.43% in moderately rocky desertified soil, and 10.06%, 42.15%, 20.66%, and 0.93% in severely rocky desertified soil, respectively. Their effects on phosphatase and catalase activities varied with the degree of rocky desertification. However, in severely rocky desertified soil, both treatments significantly increased phosphatase and catalase activities by 19.55%, 24.63%, 61.07%, and 38.05% compared to the CK, respectively. Furthermore, both electric and magnetic treatments significantly reduced bacterial α-diversity (chao1, ACE, Shannon, Simpson, and Pielou J indices) but optimized community structure by enriching dominant phyla with specific ecological functions, such as *Pseudomonadota* (7.63–41.10%), *Bacteroidota* (13.52–69.29%), and *Verrucomicrobiota* (38.26–104.81%). Functional prediction revealed that the abundances of dominant pathways (such as *chemoheterotrophy*, *aerobic chemoheterotrophy*, and *nitrogen fixation*) was enhanced following both treatments. Mantel analysis further indicated strengthened correlations among soil nutrients, enzyme activities, and bacterial communities, particularly under magnetic field treatment. These findings demonstrate that electric and magnetic field applications effectively facilitate nutrient cycling, stimulate enzyme activities, and optimize microbial community structure, thereby improving soil ecological functions and overall quality in rocky desertification regions, highlighting their potential for ecological restoration in karst areas.

## 1. Introduction

Rocky desertification is a typical form of land degradation in karst ecosystems. It is characterized by extensive exposure of the underlying bedrock due to severe loss of surface vegetation and soil, resulting in a desert-like landscape. This process is primarily driven by the synergistic effects of fragile karst geology, inappropriate human activities, and erosive rainfall [1,2]. As a widespread environmental issue affecting numerous regions globally, it underscores an urgent need for effective restoration strategies [3]. The ultimate outcome of rocky desertification is the formation of a barren rocky landscape, marked by a sharp decline in soil productivity and the degradation of ecological functions, including diminished water and nutrient retention capacity and loss of biodiversity [4]. Key manifestations include the extensive loss and disrupted cycling of essential nutrients (e.g., nitrogen, phosphorus, and potassium) [4], suppression of enzyme activities [5], and a decline in the structure and function of microbial communities [6]. These challenges are exacerbated in areas of moderate and severe rocky desertification by factors such as steeper slopes and higher rates of bedrock exposure [4]. Furthermore, the inherent geochemical properties of karst systems, such as high concentrations of Calcium ions (Ca^2+^) and Magnesium ions (Mg^2+^) and alkalinity, restrict soil nutrient desorption and bioavailability. For instance, Ca^2+^ can immobilize phosphate through precipitation, significantly reducing phosphorus availability [7]. This results in insufficient nutrient supply, thereby limiting microbial metabolic activity and further complicating restoration efforts [7]. Therefore, stimulating and enhancing the soil’s inherent biogeochemical cycling capacity is fundamental to restoring ecosystem function [8]. However, existing research has predominantly focused on controlling soil and water erosion, with few studies targeting the enhancement of nutrient levels, enzyme activities, and microbial communities in rocky desertified soils [3].

To response to these challenges, a variety of soil amelioration techniques, such as straw incorporation [9], biochar amendment [10], and mycorrhizal inoculation [11], have been developed and applied in rocky desertification areas. These methods primarily function by introducing exogenous organic materials or biological agents, which can enhance short-term soil quality to a certain extent. They have also been proven effective in improving soil structure, water retention, and fertility in many agricultural contexts. However, under the specific constraints of karst ecosystems such as shallow soil layers, high susceptibility to erosion, and unique geochemical barriers like calcium-dominated nutrient immobilization [2,7], the sustained efficacy and cost-effectiveness of these amelioration techniques may face challenges. More fundamentally, while the addition of organic materials can compensate for deficiencies in soil carbon and general nutrients, it could lead to an over-reliance on exogenous inputs for nutrient supply to soil microbes and vegetation growth. Consequently, this approach may be inadequate for effectively addressing the inherent geochemical constraints of karst environments, promoting soil biogeochemical cycling processes, and enhancing nutrient supply capacity [7]. This process critically regulates microbial activity and biogeochemical cycling. Consequently, exploring novel strategies or technologies capable of directly modulating the soil’s physicochemical microenvironment and enhancing its endogenous functional capacity remains a valuable research direction for the targeted restoration of karst ecosystems [12].

In recent years, electromagnetic field-based technologies have garnered increasing attention in environmental remediation due to their advantages of being environmentally friendly, low energy consumption, and simple operation. Electric and magnetic fields, ubiquitous in nature, play crucial roles in regulating biological processes [13,14]. Soil itself constitutes a complex electrochemical system rich in charged particles (e.g., ions, colloids), microorganisms, and their metabolites. The surface charge properties of soil particles critically influence soil physicochemical processes, such as nutrient adsorption-desorption equilibrium and aggregate stability [15]. Previous evidence suggests that exogenous electromagnetic fields may modulate the soil electrochemical environment through altering particle surface charge characteristics, ion migration, and influencing electron transfer, thereby enhancing nutrient availability, microbial metabolic activity, and enzyme synthesis [16,17]. The application of direct current to soil induces electrokinetic effects and electrochemical reactions. The electrokinetic effects involve the movement of soil water (electroosmosis), ions (electromigration), and charged particles (electrophoresis). This process promotes the transport of soil nutrients and enhances their availability. Electrochemical reactions drive water electrolysis and concurrently cause changes in environmental factors such as soil pH, water content, and temperature. These factors indirectly influence the metabolic activities of soil microorganisms [18,19]. Furthermore, the application of a magnetic field alters the magnetic environment of the soil. The generated electromagnetic waves stimulate microorganisms by affecting the chemical bonds and molecular conformations of proteins, nucleic acids, and biomolecules, thereby influencing the structural characteristics and metabolic energy of biological cells [20]. For instance, Wang et al. [21] found that an electric field of 24 V could enhance the biodegradation of polycyclic aromatic hydrocarbons in soil by regulating soil physicochemical properties and stimulating microbial activity. Zhang et al. [14] and Emamdadi et al. [22] discovered that magnetic treatment at 100–300 mT could, to some extent, enhance the nutrient availability and enzyme activities. Jin et al. [20] found that magnetic fields of 50–200 mT could improve the ecological structure and function in phosphorus tailings-based soils by stimulating microbial activity and metabolism.

Despite these advancements in electromagnetic field applications, existing research has primarily focused on the removal of organic pollutants and heavy metals from soil. However, systematic research on the improvement of rocky desertification soils quality by electromagnetic fields has not yet been reported. In particular, little is known about how soil nutrients, enzyme activities, and microbial communities in the fragile ecosystems respond to electromagnetic fields. Furthermore, existing studies have considered only single electric or magnetic field treatments, often relying on short-term pretreatment in specialized electromagnetic field devices [21,22], or employ magnetized iron powder [20] and water [23]. These methods not only overlook the potential long-term effects of sustained electromagnetic field treatment but are also prone to practical limitations: these magnetized iron powder and magnetized water are susceptible to loss through water erosion and require repeated application, increasing both cost and operational complexity. In this study, we employs continuously operational electric and magnetic field technologies to treat soils under simulated moderate and severe rocky desertification conditions. We aim to evaluate the effects and underlying mechanisms of sustained electromagnetic treatments on soil nutrient, enzyme activities, and bacterial community structure across a gradient of desertification intensity. It is expected that this work will provide important theoretical support for the rapid, efficient and low-cost remediation of soils in rocky desertification regions.

## 2. Materials and Methods

### 2.1. Experiment Device and Operation

**Figure 1:**
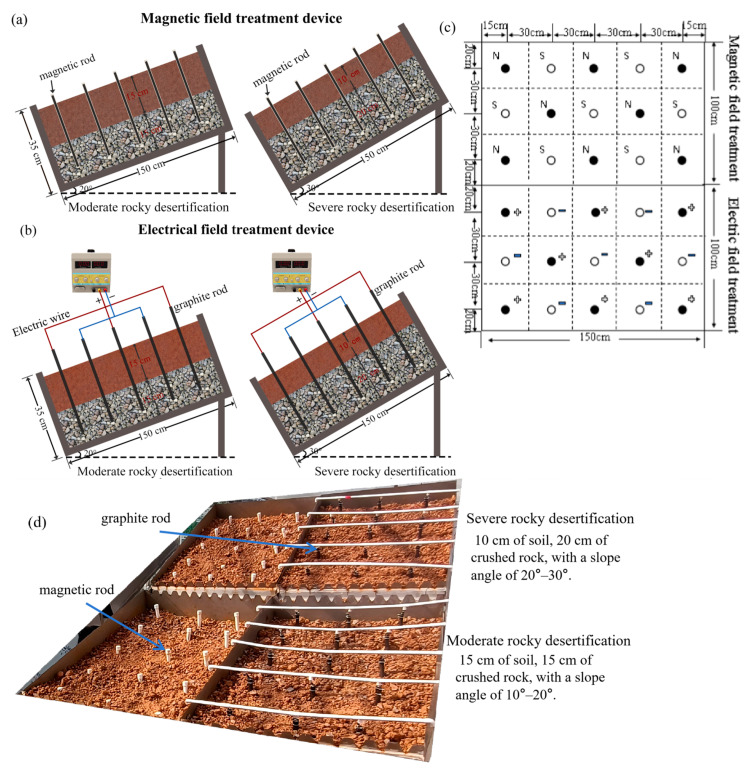
Electromagnetic field treatment device. (**a**) Magnetic field treatment; (**b**) Electrical field treatment; (**c**) Layout for Magnetic and Electric Field treatment; (**d**) Rendering of the installation;N: N-pole. S: S-pole. +: positive electrode. −: negative electrode.

The experiment was conducted in a greenhouse at the Rocky Desertification Research Institute of Southwest Forestry University. Multifunctional rocky desertification simulation tanks, independently designed by the research group, were used as experimental units; each tank measured 1.5 m × 1 m × 0.35 m (Figure 1). To realistically simulate the moderate and severe rocky desertification conditons, we adopted the classification criteria established by Jiang et al. [24], where moderate rocky desertification corresponds to a rock exposure rate of 50–70%, and severe rocky desertification exceeds 70%. Furthermore, a pre-experiment field investigation in typical rocky desertification regions of Shilin Yi Autonomous County indicated that moderate to severe rocky desertification typically occurred on slopes of 10~25°. These findings are consistent with the research results of Guo Bing et al. [25]. Following these criteria, the experimental setup simulated moderate and severe rocky desertification conditions by controlling two key factors: the soil-rock ratio and the slope gradient (simulated by tilting the experimental containers at a fixed angle) [4]. Slope gradient is a primary topographic driver in the development of rocky desertification in field conditions. It directly influences runoff velocity, soil erosion rates, and the degree of bedrock exposure, serving as a key indicator for defining the severity of rocky desertification [26]. Specifically, crushed limestone (diameter ≈ 1–2 cm) was placed at the bottom of each tank, overlain by red soil collected from Shilin Yi Autonomous County, Kunming City, a typical rocky desertification area. The moderate rocky desertification simulation group consisted of a 15 cm soil layer over 15 cm of crushed rock, with a slope angle of 20°. The severe rocky desertification simulation group comprised a 10 cm soil layer over 20 cm of crushed rock, with a slope of 30°.

For each degree of rocky desertification, three treatments were applied: electric field treatment (ET), magnetic field treatment (MT), and a control without external field (CK). Each treatment was replicated three times, resulting in a total of 18 experimental units. The field intensities were selected based on previous studies [14,21]: electric field voltage was set to 20 V, and magnetic field strength to 200 mT. Graphite electrode rods (size: 2 cm × 30 cm, Dongguan Tianwang Graphite Products Factory, Dongguan, China) were used to generate the electric field (Graphite material offers excellent electrical conductivity and is cost-effective), while stainless steel magnetic bars (size: 1.9 cm × 25 cm, Juliqiang Magnetics, Nanjing, China), sheathed in polyethylene tubes to prevent direct soil contact, were employed for magnetic field treatment. Each tank contained 15 graphite rods or magnetic bars installed 30 cm apart, with 5 cm exposed above the soil surface.

The graphite rods were connected to a constant voltage and current power supply via wires, with the voltage set at 20 V for continuous operation of the applied electric field. During the experimental period, the dry season characteristics were simulated based on the multi-year climatic features of typical rocky desertification areas in Shilin Yi Autonomous County (the area has a northern subtropical plateau mountain monsoon climate, with distinct dry and wet seasons; the dry season spans from November to April of the following year, with an average annual temperature of approximately 15 °C, and precipitation during this period accounts for only about 15% of the annual total). Soil volumetric water content was monitored daily at multiple fixed points within each experimental unit using soil moisture sensors, and was controlled to remain at 60 ± 5% of field capacity. The indoor average temperature was maintained between 15 °C and 20 °C. The experiment ran continuously for three months, from early March 2024 to late May.

### 2.2. Measurement of the Electric and Magnetic Field Strengths on the Soil Surface

**Figure 2:**
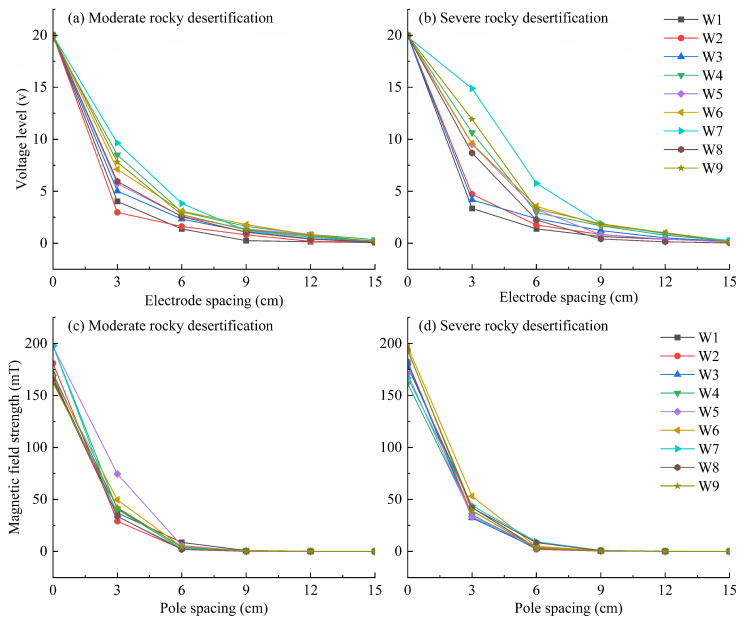
Measured variation of applied electric/magnetic field strength on the soil surface. W: Week.

After the electric and magnetic field treatments were applied, the voltage and magnetic field intensity on the soil surface were measured every 7 days. Measurements were taken at every 3 cm outward from the pole (i.e., at distances of 0, 3, 6, 9, 12, 15 cm, etc.). with measurements taken at intervals of 3 cm outward from the pole. The voltage was measured using a DC current and voltage meter S01 (WVX, CITICDA Electronics, Shenzhen, China), and the magnetic field intensity was measured using a WT103 gaussmeter (Weite Magnetoelectric Technology Co., Ltd. Shangqiu, China). As shown in Figure 2, both the voltage and the magnetic field intensity exhibited a sharp decline at a distance of 3 cm from the pole, and the field strength gradually weakened with increasing distance from the pole. The voltage approached zero at 12 cm from the pole under the electric field treatment, while the magnetic field intensity approached zero at 9 cm from the pole under the magnetic field treatment. Based on the radiation patterns of the electric and magnetic field strengths and to ensure effective monitoring of their effects on the soil microenvironment, soil sampling in this study was limited to a range of 0–10 cm from the pole.

### 2.3. Sample Collection and Analysis

#### 2.3.1. Soil Sample Collection

To investigate the effects of external field treatments on soil quality, soil samples were collected prior to the initiation of treatment and following three months of continuous field application. Within each experimental tank, sampling points were systematically distributed along an S-shaped pattern to ensure representative coverage. For the electric and magnetic field treatment groups, samples were taken within a 0–10 cm radius from the graphite rods or magnetic bars, respectively.

Soil was collected from the 0–10 cm depth. After removing roots, gravel, and other debris, soils from each sampling point were thoroughly mixed into one composite sample and bagged, and brought back to the laboratory. Each soil sample was subdivided into three parts for subsequent analyses: one part was refrigerated for determining soil enzyme activity; another was naturally air-dried in a well-ventilated, shaded area, then sieved and stored for use in soil nutrient analysis.; and the third was flash-frozen using dry ice and stored at −80 °C before being shipped to Shanghai BIOZERON Co., Ltd. (Shanghai, China) for high-throughput sequencing of soil bacteria.

#### 2.3.2. Soil Physicochemical Properties and Enzyme Activity Assay

Soil nutrient content was determined following the methods described by Bao [27]: Total nitrogen (TN) was determined using the semi-micro Kjeldahl method, total phosphorus (TP) by molybdenum-antimony anti-spectrophotometry, total potassium (TK) and available potassium (AK) by flame photometry; available nitrogen (AN) by alkaline hydrolysis diffusion method, and available phosphorus (AP) by Sodium hydrogen carbonate solution-Mo-Sb anti spectrophotometric method. Soil pH was measured using the electrode method with a water-soil ratio of 2.5:1. Soil enzyme activities were determined based on methods outlined by Guan [28]: Urease, phosphatase, sucrase, and catalase activities were determined using indophenol blue colorimetry, disodium phenyl phosphate colorimetry, 3,5-dinitrosalicylic acid colorimetry, and potassium permanganate titration, respectively.

The initial values of soil nutrients and enzyme activities in test soils with varying degrees of rocky desertification are shown in Table 1.

### 2.4. High-Throughput Sequencing of Soil Bacteria

Soil microbial community analysis was conducted through high-throughput sequencing performed by Shanghai BIOZERON Co., Ltd. (Shanghai, China). Microbial DNA was extracted from soil samples using the E.Z.N.A.^®^ Soil DNA Kit (Omega Bio-tek, Norcross, GA, USA). The hypervariable V3-V4 region of the bacterial 16S rRNA gene was amplified via polymerase chain reaction (PCR) with the primers 341 F (5′-CCTAYGGGRBGCASCAG-3′) and 806 R (5′-GGACTACNNGGGTATCTAAT-3′). The PCR protocol consisted of initial denaturation at 95 °C for 2 min; 25 cycles of denaturation at 95 °C for 30 s, annealing at 55 °C for 30 s, and extension at 72 °C for 30 s; followed by a final extension at 72 °C for 5 min. The amplification products were separated by 2% agarose gel electrophoresis and purified using the AxyPrep DNA Gel Extraction Kit (Axygen Biosciences, Union City, CA, USA). The purified PCR products were quantified using Qubit^®^ 3.0 (Life Invitrogen, Shanghai, China). DNA libraries were prepared by denaturing the PCR-amplified DNA double-stranded products and removing uncyclized DNA molecules. Sequencing was performed on the Illumina MiSeq platform using the PE300 mode. Non-repetitive sequences were clustered into operational taxonomic units (OTUs) at 97% similarity threshold using UPARSE. Chimeric sequences were identified and removed using UCHIME. Species annotation was performed using the uclust algorithm with the Silva (SSU138.2) 16S rRNA database at a confidence threshold of 80%.

### 2.5. Statistical Analysis

The changes in soil nutrient and enzyme activities were quantified as the percentage change relative to baseline values, calculated as: Rate of change = (Post-treatment value − initial value)/initial value × 100%. The experimental data were preliminarily organized using Microsoft Excel 2016. Statistical analyses were performed with IBM SPSS Statistics 27 software, covering soil nutrients, enzyme activities, and bacterial community alpha diversity, composition, and functional characteristics. Visualization plots were generated using Origin 2024 software. The Shapiro-Wilk test was employed to assess the normality of soil nutrient and enzyme activity data, and the results indicated that the data followed a normal distribution (*p* > 0.05).

One-way analysis of variance (ANOVA) was used to compare differences in soil nutrients, enzyme activities, bacterial community alpha diversity, and composition among different treatment groups under the same degree of rocky desertification. Levene’s test was applied to evaluate the homogeneity of variances among treatment groups (*p* > 0.05 indicated homogeneity of variances), followed by the LSD post-hoc test for multiple comparisons. Independent samples *t*-tests were used to assess the effects of the same treatment under different degrees of rocky desertification on soil nutrients, enzyme activities, and bacterial alpha diversity. Additionally, two-way ANOVA was conducted to examine the effects of rocky desertification degree, treatment method, and their interaction on soil nutrients and enzyme activities.

Bacterial community alpha diversity was characterized using richness indices (Chao1 index, ACE index), diversity indices (Shannon index, Simpson index), and evenness index (Pielou’s J index). Non-metric multidimensional scaling (NMDS) based on Bray-Curtis distance was performed to analyze differences in bacterial community structure among different treatment groups. Alpha diversity indices calculation and NMDS analysis were performed using the “vegan” package in R version 4.0.2, and visualizations were generated with the “ggplot 2” package. Based on bacterial OTU taxonomic information, the FAPROTAX database was utilized to predict bacterial ecological functional traits, and one-way ANOVA was used to detect differences among treatment groups. Mantel tests and multivariate statistical methods were applied to analyze the associations among soil nutrients, enzyme activities, and bacterial communities. Plots were generated using the ChiPlot platform (https://www.chiplot.online (accessed 7 October 2025)).

## 3. Results

### 3.1. Effects of External Field Treatments on Soil Nutrient Characteristics

**Figure 3:**
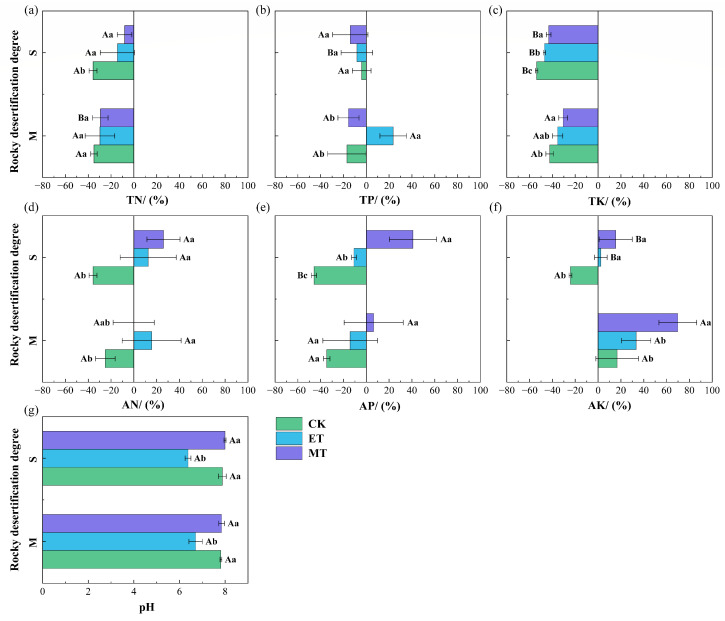
Responses of soil nutrient contents and pH to different treatments under different degrees of rocky desertification. (**a**) TN: total nitrogen; (**b**) TP: total phosphorus; (**c**) TK: total potassium; (**d**) AN: available nitrogen; (**e**) AP: available phosphorus; (**f**) AK: available potassium; (**g**) pH: soil pH. Different lowercase letters indicate significant differences among treatments within the same degree of rocky desertification (*p* < 0.05). Different capital letters indicate significant differences between different degrees of rocky desertification within the same treatment (*p* < 0.05). CK: control. ET: electric field treatment. MT: magnetic field treatment. M: moderate rocky desertification. S: severe rocky desertification.

As shown in Figure 3, in both moderately and severely rocky desertified soils, the external field treatments exerted a statistically significant influence on the changes in soil nutrients (both total and available fractions) and pH (*p* < 0.05). For the total nutrients measured (i.e., TN, TP, TK), except for total phosphorus content, the contents of total nutrients (nitrogen, potassium) in moderate and severe rocky desertification soils showed a decreasing trend under all treatments. Compared with CK, electric and magnetic field treatments effectively slowed the decline in TN and TK contents (*p* < 0.05). Specifically, in moderate rocky desertification soil, the change rates of TN and TK under the electric field treatment were −29.70% and −35.44%, respectively, both lower than those in CK (−34.96% and −42.42%), but the differences were not significant (*p* > 0.05). Under the magnetic field treatment, the change rates of TN and TK were −29.35% and −30.60%, also lower than CK, but only the difference for TK was significant (*p* < 0.05). In severe rocky desertification soil, the change rates of TN under electric and magnetic field treatments were −14.39% and −8.13%, respectively, and the change rates of TK were −46.95% and −43.30%, all significantly lower than those in the CK group (*p* < 0.05).

Compared to the decrease observed in the CK group, electric and magnetic field treatments increased the content of available nutrients in moderate and severe rocky desertification soils (*p* < 0.05), with the exception of available phosphorus under the electric field treatment. The magnetic field treatment showed a relatively better effect on enhancing available soil nutrients compared to the electric field treatment. Specifically, under the electric field treatment, the change rates of available nitrogen in moderate and severe rocky desertification soils (15.64%, 12.60%) were significantly higher than those in CK (−25.00%, −37.12%). Under the magnetic field treatment, only the severe rocky desertification soil showed a significant increase (25.92%). The contents of available phosphorus and available potassium in both moderate and severe rocky desertification soils were significantly increased under the magnetic field treatment, with their change rates being significantly higher than those in CK. Under the electric field treatment, only the values for severe rocky desertification soil were significantly higher than CK.

Regarding soil pH, the electric field treatment significantly reduced the pH in both moderate and severe rocky desertification soils compared to CK (*p* < 0.05), shifting the soil from alkaline (pH 7.69 and 7.73, respectively) to slightly acidic (pH 6.71 and 6.37, respectively). In contrast, the magnetic field treatment showed no significant effect on soil pH (*p* > 0.05).

A two-way ANOVA examining the effects of rocky desertification degree, treatment type, and their interaction on soil nutrients showed the following results (Table S1): Rocky desertification degree had a significant effect on soil total nitrogen, total potassium, and available potassium (*p* < 0.05). Treatment type significantly affected total nitrogen, available nitrogen, available phosphorus, total potassium, available potassium, and pH (*p* < 0.05). The interaction between rocky desertification degree and treatment type only had a significant effect on total phosphorus (*p* < 0.05) and showed no significant effects on the other parameters (*p* > 0.05).

### 3.2. Effects of External Field Treatments on Soil Enzyme Activity Characteristics

**Figure 4:**
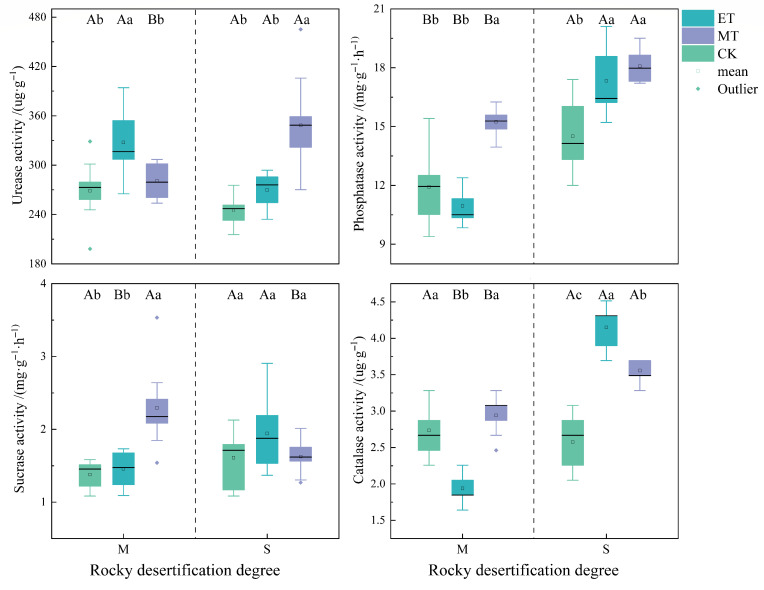
Responses of soil enzyme activities to different treatments under different degrees of rocky desertification. Different lowercase letters indicate significant differences among treatments within the same degree of rocky desertification (*p* < 0.05). Different capital letters indicate significant differences between different degrees of rocky desertification within the same treatment (*p* < 0.05). M: moderate rocky desertification. S: severe rocky desertification.

As shown in Figure 4, with the exception of sucrase activity in severely rocky desertified soil, there were statistically significant differences in soil enzyme activities among the different treatment groups (*p* < 0.05). Differences in enzyme activities were also observed between moderately and severely rocky desertified soils. Compared to the CK, both electric and magnetic field treatments increased urease activity in moderately and severely rocky desertified soils, with increases of 21.92%, 4.46%, 10.06%, and 42.15%, respectively. Notably, the enhancement reached statistical significance for the electric field treatment in moderately rocky desertified soil and for the magnetic field treatment in severely rocky desertified soil (*p* < 0.05). For phosphatase activity, in moderately rocky desertified soil, the activity under magnetic field treatment was significantly higher than that under other treatments (*p* < 0.05). In severely rocky desertified soil, the activities under both electric and magnetic field treatments were significantly higher than that in the CK (*p* < 0.05), showing respective increases of 19.55% and 24.63% compared to CK. Both electric and magnetic field treatments resulted in higher sucrase activity compared to the CK in moderately and severely rocky desertified soils, corresponding to increases of 5.70%, 66.43%, 20.66%, and 0.93%, respectively. It is noteworthy that the increase in sucrase activity under magnetic field treatment in moderately rocky desertified soil was statistically significant (*p* < 0.05). The effect on catalase activity varied with the degree of rocky desertification. Electric field treatment significantly decreased catalase activity in moderately rocky desertified soil. In contrast, both electric and magnetic field treatments significantly increased catalase activity in severely rocky desertified soil (*p* < 0.05), by 61.07% and 38.05% compared to the CK, respectively.

A two-way ANOVA further revealed (Table S1) that the interaction between the degree of rocky desertification and the treatment method had a significant effect on the activities of soil urease, phosphatase, sucrase, and catalase (*p* < 0.05).

### 3.3. Effects of External Field Treatments on Soil Bacterial Community Structure

#### 3.3.1. Soil Bacterial Community Diversity

As shown in Table 2, the analysis of soil bacterial α-diversity under different treatments and rocky desertification degrees revealed that both electric and magnetic field treatments exhibited inhibitory effects on the richness and diversity of soil bacterial communities, with no significant differences observed between moderate and severe rocky desertification soils (*p* > 0.05). Compared to CK, the richness (ACE, Chao1), diversity (Shannon, Simpson), and evenness (Pielou J) indices of soil bacterial communities in both moderate and severe rocky desertification soils under electric and magnetic field treatments were lower than those in CK. Specifically, the ACE and Chao1 indices in moderate rocky desertification soil under magnetic field treatment were significantly lower than those in CK (*p* < 0.05). In severe rocky desertification soil, the ACE and Shannon indices under both electric and magnetic field treatments, the Chao1 index under electric field treatment, and the Pielou J and Simpson indices under magnetic field treatment were significantly lower than those in CK (*p* < 0.05).

**Figure 5:**
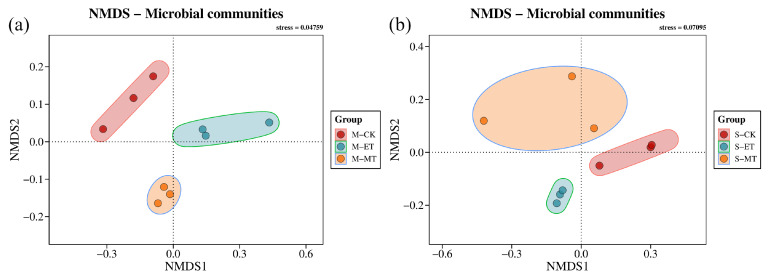
Analysis of β diversity of soil bacterial community. (**a**) moderate rocky desertification; (**b**) severe rocky desertification. M: moderate rocky desertification. CK: control. ET: electric field treatment. MT: magnetic field treatment. M: moderate rocky desertification. S: severe rocky desertification.

Non-metric multidimensional scaling (NMDS) based on Bray-Curtis distance yielded stress values of 0.04759 and 0.07095 for moderately and severely rocky desertified soils, respectively (Figure 5), both below 0.1, indicating reliable ordination. The NMDS plot revealed clear separation of soil bacteria communities by different treatments, reflecting significant differences in bacterial community composition.

#### 3.3.2. Soil Bacterial Community Composition

**Figure 6:**
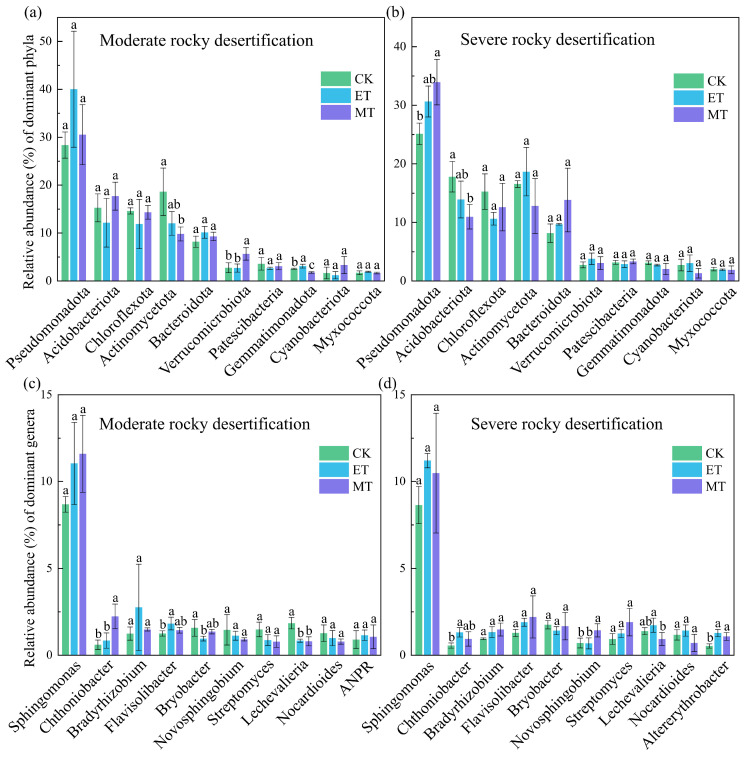
Bacterial community composition at (**a**,**b**) phylum level and (**c**,**d**) genus level. Different lowercase letters indicate significant differences among treatments within the same degree of rocky desertification (*p* < 0.05). CK: control. ET: electric field treatment. MT: magnetic field treatment. ANPR: *Allorhizobium-Neorhizobium-Pararhizobium-Rhizobium*.

The composition of bacterial community at the phylum and genus levels in moderate and severe rocky desertification soils are shown in Figure 6, respectively, with the top 10 most abundant taxa shown for each level. At the phylum level (Figure 6a,b), the dominant bacterial phyla and their relative abundances were: *Pseudomonadota* (25.13~40.02%), *Acidobacteriota* (10.96~17.81%), *Chloroflexota* (10.62~15.26%), *Actinomycetota* (9.82~18.67%), *Bacteroidota* (8.17~13.83%), *Verrucomicrobiota* (2.70~5.66%), *Cyanobacteriota* (1.20~3.34%), *Myxococcota* (1.68~2.03%), *Gemmatimonadota* (1.77~3.12%), and *Patescibacteria* (2.66~3.59%). Under moderate rocky desertification soil, the relative abundances of *Gemmatimonadota* in the electric field treatment (3.11%) was significantly higher than that in the CK group (2.56%; *p* < 0.05). Conversely, magnetic field treatment showed a significantly lower abundances of *Gemmatimonadota* (1.77%) and *Actinomycetota* (9.82%) compared to CK (2.56% and 18.62%, respectively; *p* < 0.05). Meanwhile, the relative abundances of *Verrucomicrobiota* was significantly higher under magnetic field treatment (5.66%) than under CK (2.76%; *p* < 0.05). Under severe rocky desertification soil, the magnetic field treatment exhibited a significantly higher relative abundance of *Pseudomonadota* (33.96%) compared to CK (25.13%; *p* < 0.05), while a significantly lower relative abundance of *Acidobacteriota* (10.96% vs. 17.81% in CK; *p* < 0.05).

At the genus level (Figure 6c,d), the dominant bacterial genera and their relative abundances were: *Sphingomonas* (8.64~11.59%), *Chthoniobacter* (0.60~2.24%), *Bradyrhizobium* (0.95~2.76%), *Flavisolibacter* (1.26~2.20%), *Bryobacter* (0.95~1.75%), *Novosphingobium* (0.68~1.47%), *Streptomyces* (0.78~1.91%), *Lechevalieria* (0.80~1.85%), *Nocardioides* (0.71%~1.42%), *ANPR* (in moderate desertification soil, 0.90~1.15%), and *Altererythrobacter* (in severe desertification soil, 0.54~1.28%). In moderate rocky desertification soil, the relative abundances of *Chthoniobacter* (2.24%) under magnetic field treatment and *Flavisolibacter* (1.82%) under electric field treatment were significantly higher than those in the CK group (0.60% and 1.26%, respectively; *p* < 0.05). The relative abundance of *Bryobacter* (0.95%) under electric field treatment was significantly lower than that in the CK group (1.58%; *p* < 0.05). The relative abundances of *Lechevalieria* under both electric and magnetic field treatments (0.84% and 0.80%, respectively) were significantly lower than that in the CK group (1.85%; *p* < 0.05). In severe rocky desertification soil, the relative abundances of *Chthoniobacter* (1.32%), *Lechevalieria* (1.73%), and *Altererythrobacter* (1.28%) under electric field treatment, and *Novosphingobium* (1.43%) and *Altererythrobacter* (1.08%) under magnetic field treatment were significantly higher than those in the CK group (0.55%, 1.39%, 0.54%, 0.70%, respectively; *p* < 0.05).

### 3.4. Correlation Analysis of Soil Nutrients, Enzyme Activity, and Bacterial Community Composition

**Figure 7:**
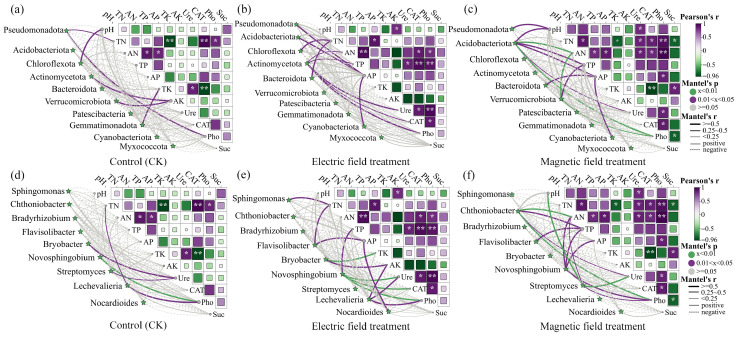
Mantel test correlations among soil nutrients, enzyme activities, and the dominant bacterial taxa at (**a**–**c**) phylumand genus (**d**–**f**) level. TN: total nitrogen. TP: total phosphorus. TK: total potassium. AN: available nitrogen. AP: available phosphorus. AK: available potassium. pH: soil pH. Ure: urease activity. CAT: catalase activity. Pho: phosphatase activity. Suc: sucrase activity. In the Mantel plot, the asterisks on the right indicate the significance level of correlations, with * representing *p* < 0.05 and ** representing *p* < 0.01.

To investigate the relationships between soil bacterial communities and environmental factors across treatments, Mantel test analysis was conducted using dominant bacterial phyla with relative abundance > 1% and the top 9 most abundant genera, along with soil nutrients and enzymes (Figure 7). Both electric and magnetic field treatments showed stronger correlations between bacterial taxa (including the dominant bacterial phyla and their subordinate taxa) and soil properties compared to CK. Under electric field treatment, *Pseudomonadota*, *Acidobacteriota*, *Chloroflexota*, *Bacteroidota*, and *Actinomycetota* showed significant relationships with soil nutrients and enzyme activities (*p* < 0.05). At the genus level, *Bryobacter* showed a highly significant positive correlation with total potassium (*p* < 0.01). *Novosphingobium* showed a significant positive correlation with total phosphorus (*p* < 0.05) and highly significant positive correlations with urease, catalase, and phosphatase (*p* < 0.01). Under magnetic field treatment, soil pH showed significant positive correlations with *Pseudomonadota* and *Acidobacteriota* (*p* < 0.05), and a highly significant positive correlation with *Streptomyces* (*p* < 0.01). Available nitrogen and phosphatase showed highly significant positive correlations with *Acidobacteriota*, *Verrucomicrobiota*, and *Chthoniobacter* (*p* < 0.01). Urease showed a highly significant positive correlation with *Streptomyces* (*p* < 0.01).

### 3.5. Potential Functions of Soil Bacterial Communities

**Figure 8:**
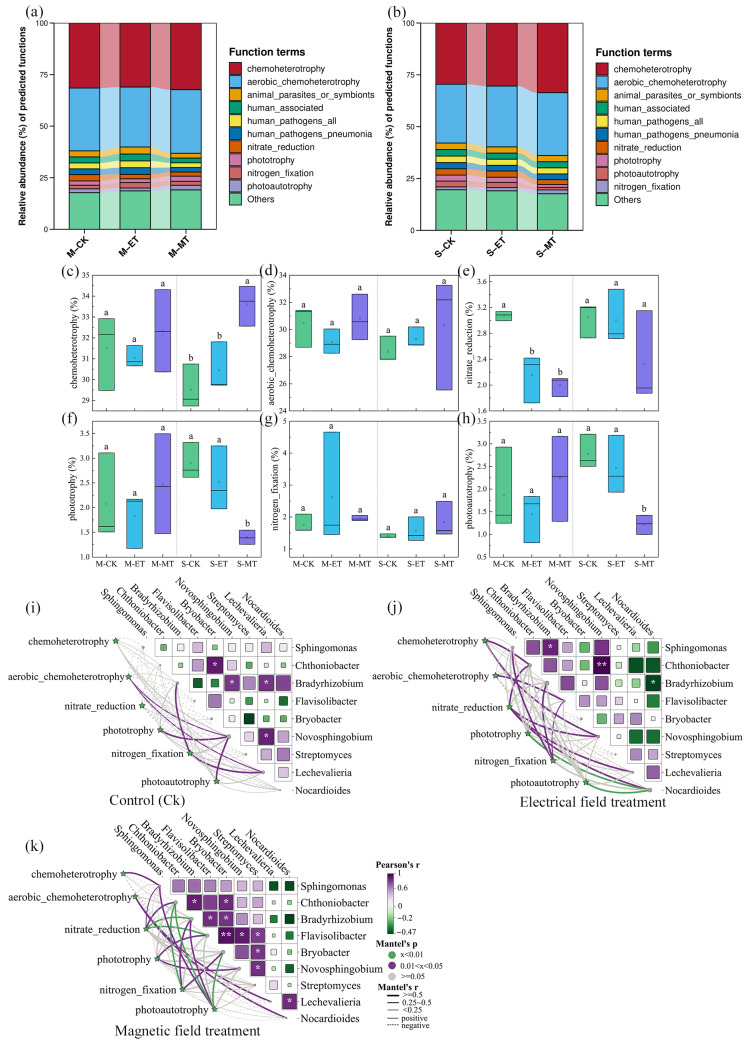
Potential functional characteristics of soil bacterial communities (**a**–**h**) and their correlation with dominant genera (**i**–**k**). (**c**–**h**): Bacterial functions related to biogeochemical cycles, different lowercase letters indicate significant differences among treatments within the same degree of rocky desertification (*p* < 0.05). CK: control. ET: electric field treatment. MT: magnetic field treatment. M: moderate rocky desertification. S: severe rocky desertification. In the Mantel plot, the asterisks on the right indicate the significance level of correlations, with * representing *p* < 0.05 and ** representing *p* < 0.01.

Based on functional annotation analysis of the soil bacterial community using the FAPROTAX database, a total of 66 functional categories were identified. The top 10 most abundant functional groups were selected for further analysis. As shown in Figure 8a,b, six of these top 10 functional groups were associated with the decomposition of organic compounds and carbon/nitrogen cycling. *Chemoheterotrophy* and *aerobic chemoheterotrophy* were identified as the predominant ecological functions of the bacterial communities in both moderate and severe rocky desertification soils. Their relative abundances under CK, electric field, and magnetic field treatments were 31.52–29.52%, 31.05–30.44%, and 32.33–33.60%, respectively. Compared to CK, the electric and magnetic field treatments resulted in higher abundances of *chemoheterotrophy* and *aerobic chemoheterotrophy*, although the differences were not statistically significant (*p* > 0.05) (Figure 8c,d). The abundance of *nitrate reduction* under electric and magnetic field treatments was significantly lower than that in CK (*p* < 0.05) in moderate rocky desertification soil, but not significantly different (*p* > 0.05) in severe rocky desertification soil. In contrast, the abundance of *nitrogen fixation* was higher under both electric and magnetic field treatments compared to CK in both moderate and severe rocky desertification soils (Figure 8g), with increases of 49.15–11.58% and 11.57–31.61%, respectively.

Mantel analysis was performed to examine the relationships between the top 9 abundant bacterial genera and key ecological functional groups. As shown in Figure 8i–k, compared to the CK group, the dominant bacterial species under electric and magnetic field treatments exhibited more pronounced roles in chemoheterotrophy, nitrogen cycling, and photosynthetic functions. Specifically, in the CK group, *Bradyrhizobium*, *Novosphingobium*, and *Lechevalieria* showed significant correlations (*p* < 0.05) with *aerobic chemoheterotrophy*, *phototrophy*, *nitrogen fixation,* and *photoautotrophy*. Under electric field treatment, *Sphingomonas*, *Chthoniobacter*, *Bradyrhizobium*, *Flavisolibacter*, *Novosphingobium*, *Lechevalieria*, and *Nocardioides* were significantly associated (*p* < 0.05) with *nitrogen fixation*, *chemoheterotrophy*, *aerobic chemoheterotrophy*, *nitrate reduction*, *phototrophy*, and *photoautotrophy*. In contrast, under magnetic field treatment, *Chthoniobacter*, *Bradyrhizobium*, *Flavisolibacter*, and *Bryobacter* demonstrated highly significant correlations (*p* < 0.01) with *nitrogen fixation*, *photoautotrophy*, and *nitrate reduction*.

## 4. Discussion

### 4.1. The Effects of External Field Treatments on Soil Nutrient Properties and Enzyme Activities

In this three-month experiment, the contents of TN, TP, and TK exhibited a decreasing trend across all treatment groups. However, the magnitude of reduction in these nutrient contents was significantly lower in the electric and magnetic field treatments compared to the control. For instance, in severely rocky desertified soil, while the CK group showed a 35.73% decrease in total nitrogen content, the electric and magnetic field treatments resulted in reductions of only 14.39% and 8.13%, respectively (Figure 3). This finding aligns with the results of Hou et al. [29], who reported that the application of a low-intensity static magnetic field reduced nitrogen loss by 29.68% during agricultural waste composting. This change may be related to the transformation of nutrient forms [30]. This point is further supported by the significant increase in the availability of nutrients (nitrogen and potassium) under electric and magnetic field treatments (Figure 3). These results indicate that external field treatments may not only inhibit nutrient loss but also promote the transformation and accumulation of available nutrients. These results may be related to processes involving ion migration and electron transfer [21,31,32]. As indicated in previous studies, charged desorbed phosphorus and potassium ions in the soil solution can migrate toward the electrodes under the influence of electromigration and electrophoresis, leading to accumulation effects [21,33]. Furthermore, electrolysis reactions can decompose soil water into H^+^ and OH^−^ ions, with H^+^ ions migrating faster than OH^−^ [31]. The resulting accumulation of H^+^ ions can significantly reduce soil pH—a phenomenon that has been observed in this study, where electric field treatment notably decreased the pH in both moderately and severely rocky desertified soils. The decrease in soil pH alters the soil environment, shifting it toward acidity. Such acidification processes are known to affect the solubility and adsorption properties of nutrients [34]. Furthermore, previous evidence also showed that the electric fields can enhance nutrient adsorption on soil colloids by altering the electrochemical properties [35]. For example, Wang et al. [36] demonstrated that enhanced soil surface charge, electrical potential, and surface electric field strength contributed to reducing NH_4_^+^ leaching and increasing total nitrogen concentration in the 0–25 cm soil layer by 46.67–61.11%. Similarly, the effect of magnetic field on enhancing nutrient availability in this study may be linked to its reported potential to increase the solubility of salts and minerals [32]. This could occur, for instance, by altering the properties of iron oxides [37], which are known to mediate crucial steps in soil nitrogen cycling [38]. Additionally, magnetic fields might also affect the adsorption-desorption equilibrium of nutrients by altering the magnetic properties of the soil, increasing soil remanence, and modifying the charge distribution [21,39]. These reported effects provide plausible explanations for the enhanced nutrient availability we measured in response to external field application, although future research directly measuring these specific mechanisms in our system is warranted.

Similar to the positive trends in soil nutrients, the external field treatments also enhanced the activity of soil enzymes associated with carbon (sucrase) cycling, nitrogen (urease) cycling, and phosphorus (phosphatase) cycling. Specifically, they promote the activities of phosphatase, sucrase, and catalase (*p* < 0.05). Accumulation of soil nutrients observed under both electric and magnetic field treatments may stimulated microbial activity, potentially contributing to the elevated enzyme activities related to carbon, nitrogen, and phosphorus cycling [40]. This study directly support this potential relationship between nutrients, microbial communities, and enzyme activity, with stronger positive correlations (*p* < 0.05) linking urease and phosphatase activities to nutrients (nitrogen availability and total phosphorus) and the abundances of key enzyme-producing microbial phyla (e.g., Pseudomonadota and Actinobacteria) under external field treatments compared to CK. Additionally, the significant associations among enzyme activities were found under external field treatments, indicating that these treatments fostered a higher degree of functional coordination within the soil microbial community regarding key biogeochemical processes. These results align with previous research proposing several pathways by which external fields might influence soil microorganisms and enzymes, such as free radicals [41], cell membrane permeability [42], or metal ion activity [43]. Notably, this study revealed that the effects of electric and magnetic fields on enzyme activities varied with the degree of rocky desertification. The electric field had a stronger effect in severely desertified soil, while the magnetic field showed better efficacy in moderately desertified soil. Existing research indicated that the surface charge density [15] and water [44] in soil decrease with the intensification of rocky desertification, which can lead to potential variations in their effects on soil enzymes [20,31]. Therefore, these differences may reflect the different application values of electric and magnetic fields for remediating soils with varying degrees of desertification due to the changes in the electrochemical properties [15] and water content [45] of the soil during the rocky desertification process, as well as plant root secretions [46].

### 4.2. The Effects of External Field Treatments on Community Structure and Function of Soil Bacterial

Changes in soil microbial diversity and community structure can reflect alterations in the soil environment, soil quality, and the progress of ecological restoration [2]. Previous studies indicated that changes in soil factors such as pH (Figure 3) [31], temperature [35], and permeability [20] induced by the application of physical fields (electric and magnetic fields) can lead to alterations in microbial activity and community composition. In this study, under both electric and magnetic field treatments, significant reductions relative to CK (*p* < 0.05) were detected for the α-diversity indices (ACE, Chao1, and, Shannon and Pielou J Indices) of soil bacterial communities. The results indicated that the environmental stress induced by external field application exerted selective pressure on bacterial communities [31]. Crucially, this shift towards lower diversity communities occurred concurrently with the significant promotion of soil nutrient accumulation and the enhancement of key biogeochemical cycling enzyme activities reported in this study. Therefore, the low-diversity communities may minimize resource competition (e.g., limitied nutrients, water, and space), favouring functionally important microorganisms [47].

Additionally, the bacterial community structure and the relative abundance of dominant phyla changed significantly, with specific ecologically functional groups occupying more important niches under stressful conditions. For example, compared to the CK group, the relative abundance of *Gemmatimonadota* (3.11%) increased significantly under electric field treatment, while *Pseudomonadota* (33.96%) and *Verrucomicrobiota* (5.66%) increased markedly under magnetic field treatment. Particularly, *Gemmatimonadota*, widely found in arid and nutrient-poor soils, possesses strong environmental adaptability and carbohydrate metabolic capacity, and its enrichment reflects enhanced carbon cycling functionality in the soil under electric field stress [48]. *Pseudomonadota*, one of the most dominant bacterial phyla in soil, includes numerous species capable of nitrogen fixation, phosphorus solubilization, and pollutant degradation; its significant enrichment suggests that magnetic field treatment may enhance the soil’s nutrient transformation capacity and ecosystem service functions [49]. Furthermore, *Pseudomonadota* frequently thrive and proliferate in eutrophic soils [50], and its increased abundance indicates a significant improvement in the quality of rocky desertified soils. *Verrucomicrobiota* participates in the degradation of various organic compounds, and its increase may help maintain the stability of soil ecological functions [51]. Significant positive correlations (*p* < 0.05) between these enriched bacterial phyla and soil nutrient availability (nitrogen, phosphorus, and potassium) support their essential contributions to nutrient cycling and soil quality improvement. Additionally, the ordination results further confirmed distinct bacterial communities across different treatments, providing strong supporting evidence that the treatments induced significant and structured shifts in communities.

Furthermore, the functional profiling revealed that *chemoheterotrophy* and *aerobic chemoheterotrophy* served as the principal ecological functions of the soil bacterial community, exhibiting higher abundances under both electric and magnetic field treatments compared to the CK group. The abundance of nitrogen fixation was also elevated under these field treatments. In contrast, the abundances of *photoautotrophy*, *nitrate reduction*, and *nitrate respiration* showed a declining trend following electric and magnetic field treatments. Given that *chemoheterotrophy* facilitates degradation of complex organic compounds [52], and *nitrogen fixation* promotes soil nitrogen cycling [53], these findings suggest that electric and magnetic field treatments may selectively enrich functional microbial taxa involved in organic matter decomposition and nitrogen cycling. Mantel analysis further demonstrated significant correlations (*p* < 0.05) between these key functions and specific bacterial genera such as *Sphingomonas* (Pseudomonadota), *Bradyrhizobium* (Pseudomonadota), and *Chthoniobacter* (Verrucomicrobiota), whose relative abundances increased under treatments. *Sphingomonas* and *Bradyrhizobium* are common nitrogen-fixing bacteria in soil, capable of establishing symbiotic relationships with various plants to facilitate nitrogen fixation [53,54]. *Chthoniobacter* is functionally involved in the degradation of complex organic compounds such as hemicellulose, lignocellulose, and cellulose, and contributes to soil carbon cycling [55]. Previous studies have demonstrated that certain bacterial phyla, including *Pseudomonadota,* *Bacteroidota*, and *Nitrospirae*, are magnetotactic and exhibit high sensitivity to electromagnetic fields, with members of these phyla showing enhanced reproduction under electromagnetic exposure [56]. This suggests that electric and magnetic field treatments may promote the proliferation of these microbial groups, thereby stimulating metabolic pathways associated with organic matter decomposition and nitrogen fixation. Such effects are of great significance for advancing biogeochemical cycling processes in rocky desertification soils, and provide support for the application of electromagnetic field technologies in maintaining soil ecosystem functional stability and enhancing restoration potential in degraded karst landscapes [13,54]. It is important to acknowledge that the inferred functional profiles presented here are based on the FAPROTAX database, representing the potential metabolic capacities of the bacterial community. To obtain a more definitive and comprehensive understanding, future research should utilize metagenomic sequencing approaches to accurately identify and quantify the functional genes and pathways modulated by electric and magnetic field applications.

Overall, electric and magnetic field treatments effectively mitigated nutrient loss and enhanced nutrient availability in rocky desertification soils. These treatments selectively reshaped bacterial communities, lowering overall α-diversity but enriching functionally important phyla (e.g., *Pseudomonadota*, *Gemmatimonadota*) involved in nutrient cycling, which positively correlated with enhanced enzyme activities (urease, phosphatase, sucrase). Our findings demonstrate that external field treatments can directly modulate soil physicochemical properties and steer microbial communities toward functional specialization, thereby enhancing biogeochemical cycling—offering a promising, targeted strategy for restoring degraded karst ecosystems.

Although this study provides preliminary evidence for the potential of electric and magnetic field treatments to improve soil quality under simulated rocky desertification conditions, several key limitations should be acknowledged. First, the experiment was conducted in a controlled greenhouse environment using small-scale soil containers, with a short operational period (three months). Moreover, only drought conditions were considered, and the stability and effectiveness of electric/magnetic field treatments for the remediation of rocky desertification soil under different climatic conditions (dry/rainy seasons) were not addressed. Therefore, the findings primarily reflect short-term responses of soil biochemical properties and microbial communities under idealized conditions. Second, the absence of plant-related indicators (e.g., biomass, root architecture, nutrient uptake) limits our ability to evaluate the actual ecological benefits of the applied treatments. Third, due to cost and feasibility constraints, this study focused exclusively on bacterial community dynamics and did not include analyses of fungi or other microorganisms. Fourth, real-time monitoring of key physicochemical parameters—such as soil redox potential, ion migration, and electrochemical properties—was not performed. Future investigations should employ advanced microsensor techniques [57] and isotope tracing [58] to quantitatively track ion movement and redox dynamics under external field exposure. Fifth, only a single field intensity was applied based on prior literature; the effects of varying field strengths remain unexplored. Future research will aim to address these limitations in order to deepen the mechanistic understanding of how electromagnetic fields influence soil ecosystems and to support the development of targeted, scalable strategies for land degradation mitigation in karst regions.

## 5. Conclusions

Electric and magnetic fields can effectively mitigate the loss rate of total nutrients in rocky desertification soil, enhance soil enzyme activities, and promote the cycling of nitrogen, phosphorus, and potassium. Both electric and magnetic field treatments reduced soil bacterial diversity, but optimized the soil bacterial community structure by selectively increasing the relative abundances of key phyla with specific ecological functions such as *Pseudomonadota*, *Bacteroidota*, *Actinomycetota*, and *Verrucomicrobiota*. This restructuring enhanced key metabolic functions within the bacterial community, particularly the *chemoheterotrophy*, *aerobic chemoheterotrophy*, and *nitrogen fixation*. These results suggest that external field remediation could be a promising approach for improving soil quality in rocky desertification areas. However, as this study was conducted under controlled greenhouse conditions over a short term and did not assess plant responses, the findings represent a preliminary proof of concept. Future field trials are necessary to evaluate the feasibility, energy efficiency, and cost-effectiveness of this technology. Furthermore, its ultimate success in accelerating ecosystem restoration must be verified by linking these soil improvements to positive plant outcomes, such as enhanced biomass, nutrient uptake, and root development.

## Figures and Tables

**Table 1:** Basic physical and chemical properties of test soils.

Index	Moderate Rocky Desertification	Severe Rocky Desertification
TN (g·kg^−1^)	2.23 ± 0.21	2.42 ± 0.61
AN (mg·kg^−1^)	24.13 ± 5.57	33.67 ± 13.47
TP (g·kg^−1^)	0.33 ± 0.05	0.33 ± 0.01
AP (mg·kg^−1^)	7.14 ± 1.45	8.65 ± 2.48
TK (g·kg^−1^)	17.35 ± 0.61	18.68 ± 0.47
AK (mg·kg^−1^)	61.48 ± 4.16	74.93 ± 9.41
pH	7.69 ± 0.04	7.73 ± 0.07
Ure (ug·g^−1^)	429.91 ± 90.48	569.84 ± 160.94
CAT (ug·g^−1^)	1.25 ± 0.28	2.83 ± 1.69
Pho (mg·g^−1^·h^−1^)	12.05 ± 3.48	13.63 ± 4.5
Suc (mg·g^−1^·h^−1^)	0.55 ± 0.11	0.55 ± 0.09

TN: total nitrogen. TP: total phosphorus. TK: total potassium. AN: available nitrogen. AP: available phosphorus. AK: available potassium. pH: soil pH. Ure: urease activity. CAT: catalase activity. Pho: phosphatase activity. Suc: sucrase activity.

**Table 2:** Analysis of α-diversity of soil bacterial communities.

Treatment	ACE Index	Chao1 Index	Shannon Index	Pielou J Index	Simpson Index
M-CK	4168.48 ± 125.78 Aa	4020.43 ± 133.22 Aa	6.64 ± 0.14 Aa	0.8296 ± 0.0130 Aa	0.996 4 ± 0.0007 Aa
M-ET	3809.29 ± 281.31 Aab	3748.15 ± 212.20 Aab	6.56 ± 0.20 Aa	0.8262 ± 0.0156 Aa	0.9961 ± 0.0009 Aa
M-MT	3717.77 ± 66.11 Ab	3628.85 ± 49.02 Ab	6.46 ± 0.08 Aa	0.8172 ± 0.0075 Aa	0.9955 ± 0.0005 Aa
S-CK	4181.94 ± 205.20 Aa	4117.49 ± 205.51 Aa	6.81 ± 0.13 Aa	0.8473 ± 0.0097 Aa	0.9972 ± 0.0004 Aa
S-ET	3593.38 ± 138.76 Ab	3538.24 ± 134.89 Ab	6.58 ± 0.04 Ab	0.8326 ± 0.0035 Aab	0.9963 ± 0.0003 Aab
S-MT	3817.50 ± 160.96 Ab	3778.29 ± 184.69 Aab	6.56 ± 0.11 Ab	0.8257 ± 0.0109 Ab	0.9957 ± 0.0011 Ab

Different lowercase letters indicate significant differences among treatments within the same degree of rocky desertification (*p* < 0.05). Different capital letters indicate significant differences between different degrees of rocky desertification within the same treatment (*p* < 0.05). M: moderate rocky desertification. S: severe rocky desertification. CK: control. ET: electric field treatment. MT: magnetic field treatment.

## Data Availability

The original contributions presented in this study are included in the article/supplementary material. Further inquiries can be directed to the corresponding authors.

## Supplementary Materials

The following supporting information can be downloaded at: https://www.mdpi.com/article/10.3390/microorganisms14040934/s1, Figure S1: RDA analysis of the relationship between soil environmental factors and bacterial community composition at the phylum level. (a) control; (b) electric field treatment; (c) magnetic field treatment; (d) overall RDA analysis. TN: total nitrogen. TP: total phosphorus. TK: total potassium. AN: available nitrogen. AP: available phosphorus. AK: available potassium. pH: soil pH. Ure: urease activity. CAT: catalase activity. Pho: phosphatase activity. Suc: sucrase activity. Figure S2: RDA analysis of the relationship between soil environmental factors and bacterial community composition at the genus level. (a) control; (b) electric field treatment; (c) magnetic field treatment; (d) overall RDA analysis. TN: total nitrogen. TP: total phosphorus. TK: total potassium. AN: available nitrogen. AP: available phosphorus. AK: available potassium. pH: soil pH. Ure: urease activity. CAT: catalase activity. Pho: phosphatase activity. Suc: sucrase activity. Table S1: Analysis of rocky desertification degree and treatment methods and their interaction. Degree: rocky desertification degree. Treatment: electric field treatment and magnetic field treatment. TN: total nitrogen. AN: available nitrogen. TP: total phosphorus. AP: available phosphorus. TK: total potassium. AK: available kalium . pH: soil pH. Table S2: Comparison of differences in soil nutrient content and enzyme activity before and after treatment. CK: control. ET: electric field treatment. MT: magnetic field treatment. Table S3: Energy consumption parameters of the electric field treatment. M: moderately rocky desertification soil; S:severely rocky desertification soil. U: voltage; I: current; t: treatment duration; Q: energy consumption.
