# Supplementary material for: Effects of Continuous Electric/Magnetic Field Treatment on Nutrient, Enzyme Activity, and Bacterial Community Structure in Rocky Desertification Soils
Source: Microorganisms. 2026 Apr 21;14(4):934. doi: 10.3390/microorganisms14040934 (PMC13119125; doi:10.3390/microorganisms14040934)
Supplement: Supplementary file 1 [file microorganisms-14-00934-s001.zip › microorganisms-4230116-supplementary.pdf]

Supplementary data

A two-way ANOVA examining the effects of rocky desertification degree, treatment type, and their interaction on soil nutrients showed the following results (Table S1): Rocky desertification degree had a significant effect on soil total nitrogen, total potassium, and available potassium ($P < 0.05$). Treatment type significantly affected total nitrogen, alkaline-hydrolyzable nitrogen, available phosphorus, total potassium, available potassium, and pH ($P < 0.05$). The interaction between rocky desertification degree and treatment type only had a significant effect on total phosphorus ($P < 0.05$) and showed no significant effects on the other parameters ($P > 0.05$).

A two-way ANOVA further revealed (Appendix.) that the interaction between the degree of rocky desertification and the treatment method had a significant effect on the activities of soil urease, phosphatase, sucrase, and catalase ($P < 0.05$). This indicates that the effects of external field treatments on soil enzyme activities were influenced by the degree of rocky desertification.

Table S1. Analysis of rocky desertification degree and treatment methods and their interaction

Type	Index	Variation source	df	F	P
Soil Nutrients	TN	Degree	1	7.683	0.017
		Treatment	2	5.569	0.019
		Degree × Treatment	2	2.334	0.139
	AN	Degree	1	0.152	0.704
		Treatment	2	10.107	0.003
		Degree × Treatment	2	1.521	0.258
	TP	Degree	1	0.892	0.364
		Treatment	2	5.107	0.025
		Degree × Treatment	2	4.833	0.029
	AP	Degree	1	1.268	0.282
		Treatment	2	21.925	<0.001
		Degree × Treatment	2	2.891	0.094
	TK	Degree	1	72.922	<0.001
		Treatment	2	21.979	<0.001
		Degree × Treatment	2	0.086	0.918
	AK	Degree		46.585	<0.001
		Treatment		18.892	<0.001
		Degree × Treatment		1.21	0.332
	pH	Degree	1	1.65	0.223
		Treatment	2	151.058	<0.001
		Degree × Treatment	2	3.531	0.062
Soil enzymes	Urease activity	Degree	1	0.23	0.634
		Treatment	2	12.049	<0.001
		Degree × Treatment	2	14.309	<0.001
	Phosphatase activity	Degree	1	110.647	<0.001
		Treatment	2	30.317	<0.001
		Degree × Treatment	2	10.793	<0.001
	Sucrase activity	Degree	1	0.022	0.883
		Treatment	2	6.77	0.003

Type	Index	Variation source	df	F	P
		Degree × Treatment	2	11.457	<0.001
	Catalase activity	Degree	1	138.303	<0.001
		Treatment	2	21.129	<0.001
		Degree × Treatment	2	85.243	<0.001

Degree: rocky desertification degree. Treatment: electric field treatment and magnetic field treatment. TN: total nitrogen. AN: available nitrogen. TP: total phosphorus. AP: available phosphorus. TK: total potassium. AK: available kalium . pH: soil pH.

The differences in soil nutrient content and enzyme activity before and after treatment are shown in the table below:

Table S2. Comparison of differences in soil nutrient content and enzyme activity before and after treatment

Index		Moderate rocky desertification			Severe rocky desertification		
		CK	ET	MT	CK	ET	MT
TN	Initial value	2.48±0.09	2.14±0.1	2.06±0.09	3.17±0.15	1.83±0.25	2.25±0.09
/（$g{\cdot}kg^{-1}$）	Processed value	1.61±0.02	1.50±0.21	1.45±0.09	2.03±0.06	1.55±0.12	2.07±0.11
AN	Initial value	30.86±4.11	21.34±0	20.19±2.06	51.09±2.06	26.17±4.12	23.76±4.12
/（$mg{\cdot}kg^{-1}$）	Processed value	23.11±3.66	24.68±5.51	19.90±1.38	32.34±12.15	28.82±2.07	29.52±1.39
TP	Initial value	0.39±0.07	0.31±0	0.3±0	0.34±0.01	0.32±0.01	0.32±0
/（$g{\cdot}kg^{-1}$）	Processed value	0.32±0.01	0.39±0.03	0.25±0.03	0.32±0.02	0.30±0.05	0.27±0.05
AP	Initial value	8.07±0.46	8.1±0.36	5.26±0.36	11.37±0.71	8.8±0.84	5.78±0.08
/（$mg{\cdot}kg^{-1}$）	Processed value	5.26±0.08	6.91±1.76	5.54±0.98	6.17±0.65	7.85±0.79	8.16±1.24
TK	Initial value	17.67±0.88	16.95±0.28	17.44±0.5	19.18±0.42	18.57±0.28	18.29±0.01
/（$g{\cdot}kg^{-1}$）	Processed value	10.16±0.25	10.93±0.61	12.11±0.94	8.84±0.06	9.85±0.07	10.37±0.38
AK	Initial value	60.7±1.18	66.4±0.59	57.35±2.12	85.56±1.02	63.9±0.59	75.35±1.02
/（$mg{\cdot}kg^{-1}$）	Processed value	70.98±12.77	88.60±9.06	97.58±12.03	64.84±1.45	65.41±3.62	86.95±10.64
pH	Initial value	7.7±0.01	7.65±0.05	7.7±0.01	7.8±0	7.65±0.05	7.75±0.05
	Processed value	7.81±0.04	6.71±0.30	7.84±0.13	7.88±0.17	6.37±0.12	7.99±0.05
Ure	Initial value	499.86±21.6	467.5±31.18	322.36±67.07	732.85±94.92	388.61±11.7	588.06±69.6
/（$ug{\cdot}g^{-1}$）	Processed value	268.82±17.26	327.75±42.84	280.83±21.52	245.07±4.78	269.72±10.53	348.36±31.67
CAT	Initial value	1.16±0.12	1.09±0.24	1.5±0.31	4.99±0.12	1.23±0	2.26±0.36
/（$ug{\cdot}g^{-1}$）	Processed value	2.74±0.21	1.94±0.22	2.94±0.25	2.58±0.39	4.15±0.27	3.56±0.15
Pho	Initial value	16.22±0.39	11.69±0.1	8.24±0.52	18.98±1.42	8.77±0.24	13.15±0.65
/（$mg{\cdot}g^{-1}{\cdot}h^{-1}$)	Processed value	11.92±1.88	10.95±0.95	15.24±0.65	14.50±1.78	17.34±1.62	18.08±0.82
Suc	Initial value	0.65±0.01	0.59±0.02	0.4±0.01	0.66±0.02	0.48±0.03	0.51±0.04
/（$mg{\cdot}g^{-1}{\cdot}h^{-1}$)	Processed value	1.38±0.19	1.46±0.18	2.29±0.21	1.61±0.42	1.94±0.40	1.62±0.25

CK: control. ET: electric field treatment. MT: magnetic field treatment.

Redundancy analysis (RDA) was further employed to identify the key soil physicochemical factors influencing the dominant bacterial phyla. As shown in Fig. S1, in the CK group, the first two axes cumulatively explained 78.63% of the total variation, with soil available potassium, sucrase, total nitrogen, phosphatase, and pH identified as the main factors influencing bacterial community composition. Under electric field treatment, the first two axes accounted for 88.75% of the total variation, with soil available phosphorus, phosphatase, and sucrase showing positive correlations with Actinomycetota and Cyanobacteriota, and serving as the primary factors shaping community composition. Under magnetic field treatment, the first two axes explained 84.06% of the total variation, with soil total nitrogen, alkali-hydrolyzable nitrogen, available phosphorus, phosphatase, and sucrase emerging as the dominant influencing factors. In the overall RDA (without treatment grouping), the first two axes cumulatively explained 60.27% of the total variation, with soil total nitrogen, available potassium, pH, phosphatase, and catalase identified as the key factors affecting bacterial community composition.

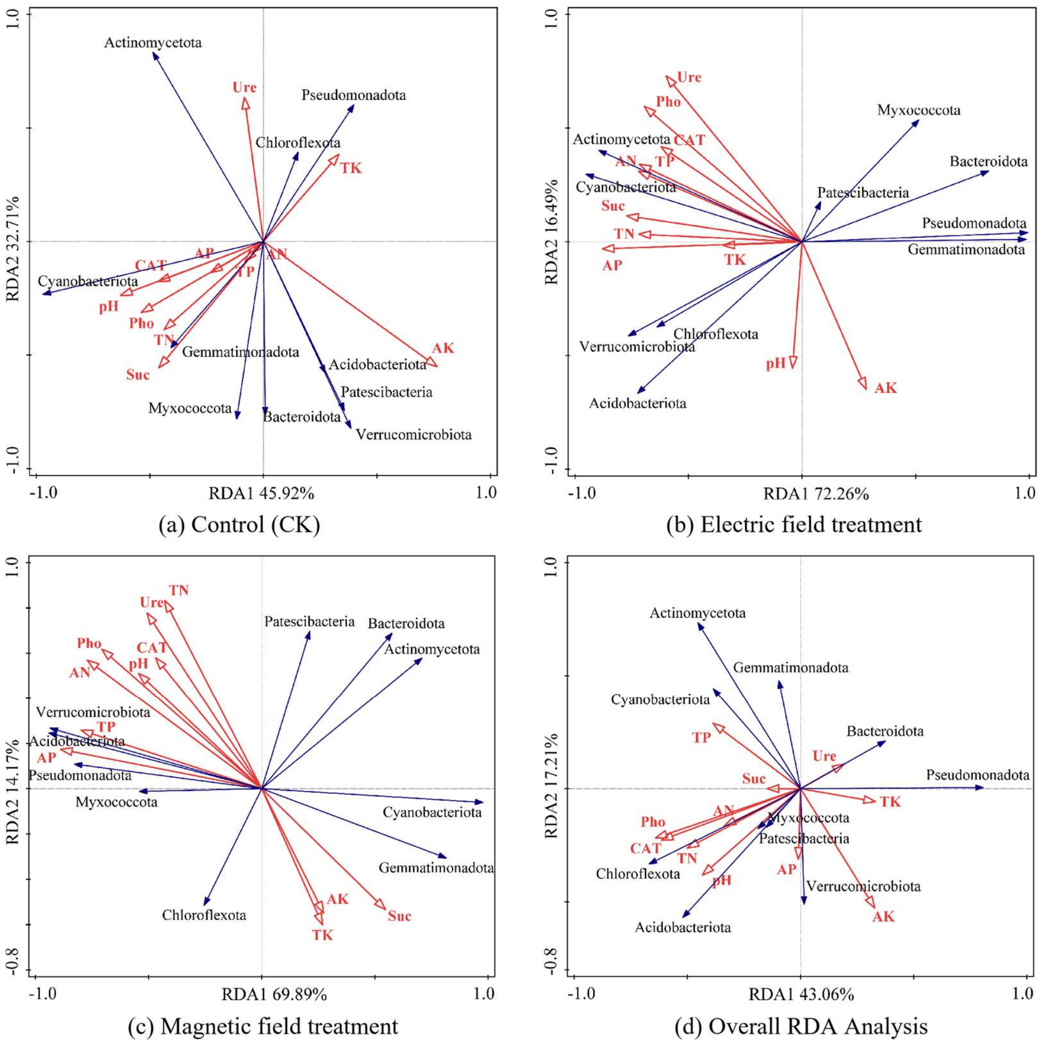

Figure S1. RDA analysis of the relationship between soil environmental factors and bacterial community composition at the phylum level. TN: total nitrogen. TP: total phosphorus. TK: total potassium. AN: available nitrogen. AP: available phosphorus. AK: available potassium. pH: soil pH. Ure: urease activity. CAT: catalase activity. Pho: phosphatase activity. Suc: sucrase activity.

Redundancy analysis (RDA) was further performed to identify the key soil physicochemical factors

influencing the dominant bacterial genera. As shown in Fig. S2, in the CK group, the first two axes cumulatively explained 86.06% of the total variation. Soil available potassium was positively correlated with *Chthoniobacter*, *Flavisolibacter*, and *Bryobacter*, while soil urease, total potassium, and pH showed positive correlations with *Sphingomonas*, *Bradyrhizobium*, *Streptomyces*, *Lechevalieria*, and *Nocardioides*. Under electric field treatment, the first two axes accounted for 88.10% of the total variation, with soil pH negatively correlated with *Sphingomonas*, *Bradyrhizobium*, *Flavisolibacter*, *Streptomyces*, and *Lechevalieria*. Under magnetic field treatment, the first two axes explained 78.34% of the total variation. Soil total phosphorus, available phosphorus, alkali-hydrolyzable nitrogen, pH, and urease were positively correlated with *Flavisolibacter*, *Novosphingobium*, *Streptomyces*, and *Bryobacter*. In the overall RDA (without treatment grouping), the first two axes cumulatively explained 54.20% of the total variation, with soil available phosphorus, total nitrogen, pH, phosphatase, catalase, and urease identified as the primary factors influencing bacterial community composition.

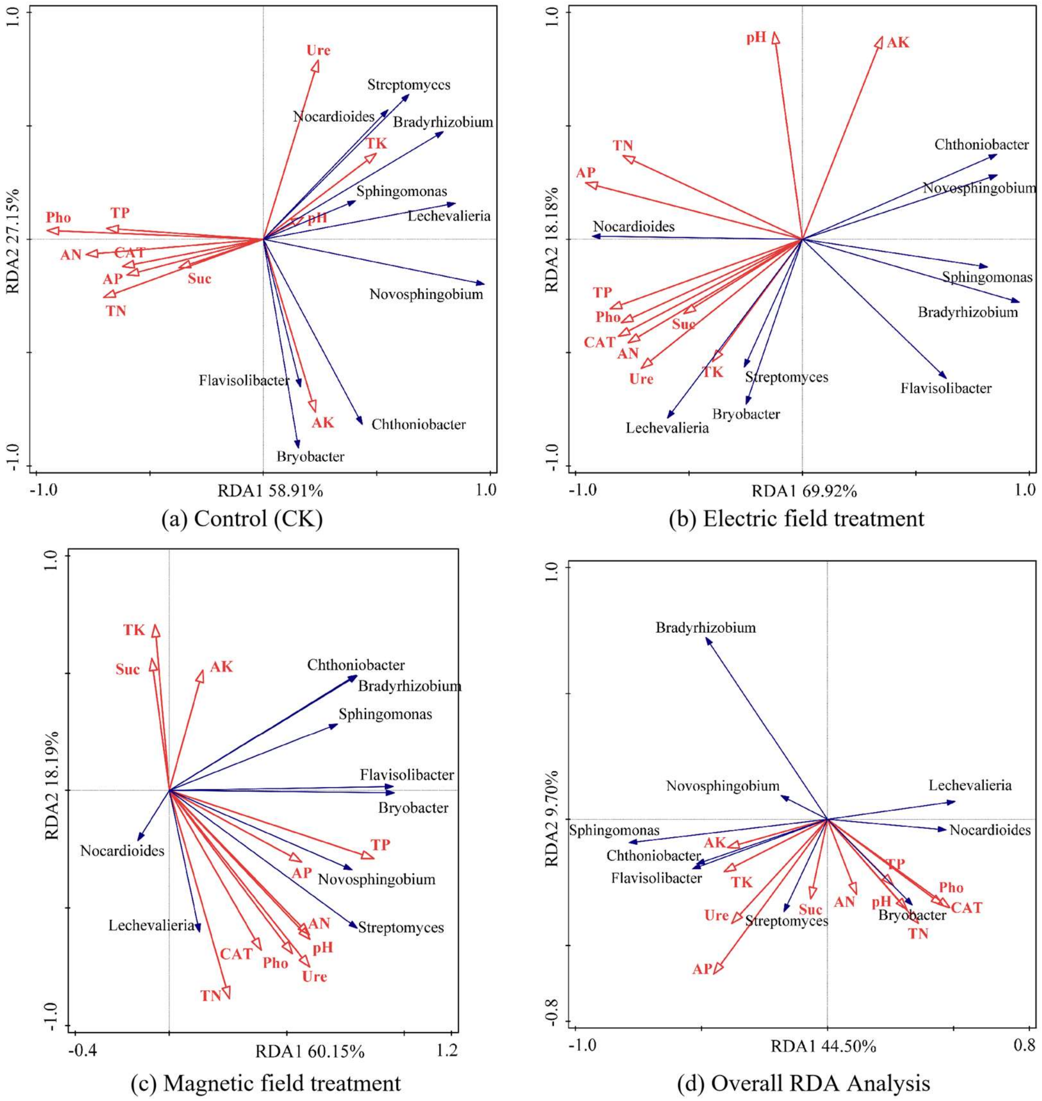

Figure S2. RDA analysis of the relationship between soil environmental factors and bacterial community composition at the genus level. TN: total nitrogen. TP: total phosphorus. TK: total potassium. AN: available nitrogen. AP: available phosphorus. AK: available potassium. pH: soil pH. Ure: urease activity. CAT: catalase activity. Pho: phosphatase activity. Suc: sucrase activity.

Energy consumption comparison

Referring to the equation used by Wang, Sa et al., the energy consumption of the electric field treatment was calculated using the following formula:

$$Q = U \times I \times t$$

where Q is the energy consumption (kW·h), U is the voltage (V), I is the current (mA), and t is the duration of the electric field treatment (h).

The energy consumption parameters for the electric field treatment in moderately and severely rocky desertification soils are presented in Table S3. The applied voltage was 20 V in both cases, with a total duration of 2208 h (3 months). In the moderately rocky desertification soil, the current ranged from 16 to 80 mA, and the total energy consumption was 1.8216 kW·h. In the severely rocky desertification soil, the current ranged from 22 to 93 mA, and the total energy consumption was 2.34056 kW·h. These results indicate that the current in the severely rocky desertification soil was generally higher than that in the moderately rocky desertification soil, leading to greater total energy consumption, which may be related to differences in ion concentration or soil electrochemical properties between the two degrees of desertification.

Table S3 Energy consumption parameters of the electric field treatment

Degree	U (V)	I (mA)	t (h)	Q (kw·h)
M	20	16-80	2208	1.8216
S	20	22-93	2208	2.3405

M: moderately rocky desertification soil; S:severely rocky desertification soil.

Reference: Wang, Sa, et al. Insights into electro-bioremediation of PAH-contaminated soil under polarity reversal conditions: Effect of effective current intensity and soil properties on microbial function..Chemical Engineering Journal. 2023, 478: 147493.
